# The fully engaged inspiratory muscle training reduces postoperative pulmonary complications rate and increased respiratory muscle function in patients with upper abdominal surgery: a randomized controlled trial

**DOI:** 10.1080/07853890.2022.2106511

**Published:** 2022-08-09

**Authors:** Yu-Ting Huang, Yih-Jyh Lin, Ching-Hsia Hung, Hui-Ching Cheng, Hsin-Lun Yang, Yi-Liang Kuo, Pei-Ming Chu, Yi-Fang Tsai, Kun-Ling Tsai

**Affiliations:** a Department of Physical Therapy, College of Medicine, National Cheng Kung University, Tainan, Taiwan; b Department of Surgery, National Cheng Kung University Hospital, College of Medicine, National Cheng Kung University, Tainan, Taiwan; c Institute of Allied Health Sciences, College of Medicine, National Cheng Kung University, Tainan, Taiwan; d Department of Anatomy, School of Medicine, Chung Shan Medical University, Taichung, Taiwan; e Department of Medical Education, Chung Shan Medical University Hospital, Taichung, Taiwan

**Keywords:** Upper abdominal surgery, Inspiratory muscle training, Cardiopulmonary function, Diaphragm ultrasonography, Postoperative pulmonary complications

## Abstract

**Background:**

Upper abdominal surgical treatment may reduce respiratory muscle function and mucociliary clearance, which might be a cause of postoperative pulmonary complications (PPCs). Threshold inspiratory muscle training (IMT) may serve as an effective modality to improve respiratory muscle strength and endurance in patients. However, whether this training could help patients with upper abdominal surgery remains to be determined. The aim of the present investigation was to determine the effect of a fully engaged IMT on PPCs and respiratory function in patients undergoing upper abdominal surgery. We hypothesized that the fully engaged IMT could reduce PPCs and improve respiratory muscle function in patients with upper abdominal surgery.

**Methods:**

This is a randomized controlled trial (RCT) with 28 patients who underwent upper abdominal surgery. Patients were randomly assigned to the control (CLT) group or the IMT group. The CTL group received regular health care. The IMT group received 3 weeks of IMT with 50% of MIP as the initial intensity before the operation. The intensity of MIP increased by 5–10% per week. The IMT was continued for 4 weeks after the operation. The study investigated the outcomes including PPCs, respiratory muscle strength, diaphragmatic function, cardiopulmonary function, and quality of life (QoL).

**Results:**

We found that IMT improved respiratory muscle strength and diaphragmatic excursion. IMT also had a beneficial effect on the incidence of postoperative pulmonary complications (PPCs) compared to CLT care.

**Conclusion:**

The results from this study revealed that IMT provided positive effects on parameters associated with the respiratory muscle function and reduced the incidence of PPCs. We propose that fully engaged IMT should be a part of clinical management in patients with upper abdominal surgery.

**KEY MESSAGES**
- The fully engaged inspiratory muscle training reduces postoperative pulmonary complications rate in patients with upper abdominal surgery.
- The fully engaged inspiratory muscle training increases maximal inspiratory pressure in patients with upper abdominal surgery.
- The fully engaged inspiratory muscle training increases diaphragm function in patients with upper abdominal surgery.
- The fully engaged inspiratory muscle training increases the quality of life in patients with upper abdominal surgery.

## 1. Introduction

Upper abdominal surgery is the most common major surgery [1,2]. A major surgery under general anaesthesia causes a rapid decline in functional residual capacity (FRC) of up to 20% and impairs the normal activity of respiratory muscle groups, especially in the diaphragm [3]. Respiratory muscle dysfunction may reduce lung capacity, tidal volume, and coughing function. FRC and mucociliary clearance are likely to decrease. As a result, atelectasis in the base of the lung segment, diaphragm palsy, infection and hypoxia might develop [4]. The impairment of respiratory function after an operation is collectively referred to as postoperative pulmonary complications (PPCs) [1–3,5–7]. It is estimated that the frequency of PPCs after major surgical procedures ranges from 1 to 30% of the patients. In most cases, the risk of PPCs increases with the distance from the surgical site to the diaphragm [1,3]. Due to this unique physiological mechanism, the risk of PPCs after upper abdominal incision surgery may be up to 15 times higher than that after lower abdominal incision surgery [3]. PPCs increase the length of hospital stay, mortality rate, and medical consumption. It is even the main cause of postoperative death [1,2,7–11].

The training of diaphragm breathing increases the diaphragm excursion, thereby improving the inflation of alveoli and ventilation, reducing the chance of hypoxaemia and the increased work of breathing [12]. Diaphragm dysfunction is one of the causes of PPCs. Traditional chest physical therapy has been widely used to prevent the development of PPCs in patients undergoing upper abdominal surgery. However, the incidence of PPCs remains high [13]. Threshold inspiratory muscle training (IMT) was found to be more effective in increasing muscle strength than the incentive spirometer and deep breath exercise [14]. Since the 1980s, threshold IMT has been widely used as a non-pharmacological intervention to enhance inspiratory muscle strength for the management of respiratory symptoms [6]. The purpose of IMT is to improve the strength and endurance of inspiratory muscles. Resistance is used in IMT to exercise respiratory muscles [6,15,16]. However, whether IMT has beneficial effects on a patient with upper abdominal surgery is still unclear.

The main purpose of our study was to investigate the effect of intervening threshold IMT in patients undergoing upper abdominal surgery. We provided a comprehensive IMT program encompassing from preoperative to postoperative periods. We assessed cardiopulmonary function, incidence of PPCs, changes in diaphragm echography, respiratory muscle strength, and quality of life (QoL) in patients undergoing upper abdominal surgery.

## 2. Materials and methods

### 2.1. Study participants

We conducted a parallel randomized controlled trial (RCT) between April 2019 and December 2020 at a single-center, tertiary hospital in Tainan, Taiwan. This randomized control trial conformed to the CONSORT statement (Supplementary material Appendix 1) and was registered in the Thai Clinical Trials Registry (TCTR20190526001) and ClinicalTrials.gov (NCT05239819). This study was also approved by the ethics committee of the National Cheng Kung University Hospital Institutional Review Board, Tainan, Taiwan (B-BR-108-012). After explaining all the experimental procedures in detail, each eligible participant provided written consent to join the study. The participants for this study were recruited from the Department of General Surgery at National Cheng Kung University Hospital (NCKUH), Tainan, Taiwan. We enrolled 28 patients with upper abdominal surgery. The inclusion criteria of this study were as follows: (1) ≥20 years old with upper abdominal surgery, (2) American Society of Anaesthesiologists (ASA) I-IV, (3) body mass index (BMI) ≥ 18, and (4) able to follow the exercise protocol. The exclusion criteria of this study were as follows: (1) history of prior abdominal surgery, (2) exercise contraindications due to high risk (e.g. severe cardiac or cardiovascular disease), (3) American Society of Anaesthesiologists; ASA V-IV, (4) unable to follow the exercise protocol and (5) severe organ failure. The online random generator was used for randomization. Blinding was not achievable both in subjects and researchers due to an individualized respiratory muscle training program.

### 2.2. Procedures

**Figure 1.**
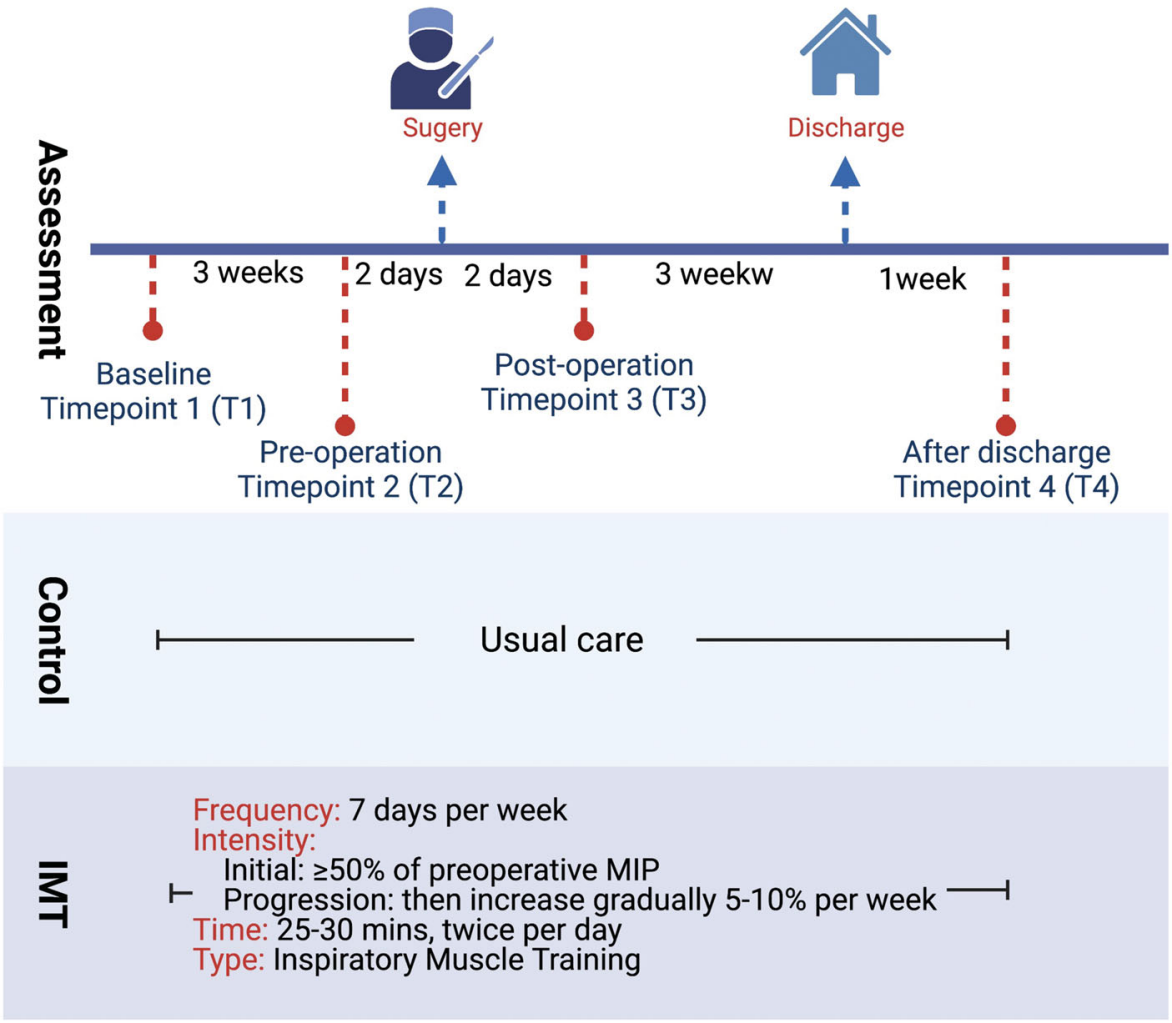
The schematic outline of this study and time points of outcome measurements.

After the participants signed the informed consent form, the same baseline measurements were made in all enrolled patients. They were assigned randomly to the control group or the exercise group. We generated a list of allocation sequences by using computer-generated code. The allocation was concealed, but the collection of data was not blinded in this study. The measurements were made 3 weeks before operation (baseline; Timepoint 1), 2 days before operation (pre-operation; Timepoint 2), 2 days after operation; (post-operation; Timepoint 3), and 4 weeks after the operation (1 week after discharge; Timepoint 4). Figure 1 shows the outline of the current study and time points of outcome measurements.

### 2.3. Intervention

We conducted a fully engaged inspiratory muscle training (IMT) program. The exercise group received the intervention threshold IMT before and after the upper abdominal surgery. The IMT was started 3 weeks before the operation and continued for the next 4 weeks. The initial training intensity of the preoperative threshold IMT trainer (DofinTM, Breathing Strength Builder, Taiwan) was moderate to high (≥50% of MIP), which was determined according to the patient’s baseline level, and the intensity was increased by 5–10% per week. The duration of the training was 25–30 min each time, twice a day, and five days per week for at least two weeks. The participants received at least 10 training sessions before surgery. When removing the endotracheal tube after surgery, the threshold IMT was applied immediately. Then, the training intensity of IMT was set based on the measurement of MIP after the operation. The training program was maintained for 25–30 min each time, twice a day and five days per week, until discharge (Figure 1).

### 2.4. Outcome measures

Socio-demographic and medical characteristics, including age, sex, and diagnosis, were recorded at baseline. The primary outcomes investigated in association with the incidence of PPCs were respiratory muscle strength (maximum inspiratory pressure (PImax or MIP), maximum expiratory pressure (PEmax or MEP)), cardiopulmonary function tests (CPET), diaphragm excursion, and thickening fraction by ultrasound. The secondary outcomes were the pulmonary function test, length of hospital stay, and QoL.

#### 2.4.1. Postoperative pulmonary complication rate (PPC rate)

We used the criteria in the definition of PPCs in European Perioperative Clinical Outcome 2015 [17] to calculate the incidence of PPCs in this study. Participants received blood biochemistry analysis, bacterial culture, and chest X-ray at least weekly after surgery until discharge. Each abnormal finding was counted as the incidence of a unique adverse outcome based on the diagnosis of the clinician and pathologist.

#### 2.4.2. Respiratory muscle strength

MIP and MEP were determined by measuring the upper airway pressure during maximum voluntary inspiration and expiratory. MIP was usually measured after maximal exhalation when approaching residual volume (RV). MEP was measured at or near-total lung capacity (TLC). According to the guidelines of the American Thoracic Society (ATS)/European Respiratory Society (ERS), the standard way is to let participants take a sitting posture wearing a nose clip, bite the mouthpiece and hold the pressure gauge with one hand while the other hand fixes the lips with fingers to avoid air leakage. Maximum inhalation and exhalation are performed against a gas pressure gauge with a one-way valve. The measurements are repeated 3 times, taking the maximum value as a record [18,19]. The task was performed at baseline, pre-operation, post-operation, and one week after discharge.

#### 2.4.3. Cardiopulmonary exercise test (CPET)

We used the Bruce ramp protocol and ACSM suggestions to perform the maximal exercise test. The CPET was performed with the cycle ergometer (Ultima CPX, MGC, US). Metabolic data were collected from expired gas breath-by-breath. The test started at a low workload and the load was progressively increased according to a ramp protocol with increments of 20 W every minute under a consistent pedalling speed of approximately 50–60 rpm for 6 to 15 min. The CPET was terminated when the subject reached the maximal exercise intensity or limiting symptoms. The task was performed at baseline and one week after discharge.

#### 2.4.4. Diaphragm ultrasonography

The diaphragm mobility and thickness were evaluated by diaphragm ultrasonography. The excursion at the dome of the diaphragm and the muscle thickening at the zone of apposition (ZOA) were measured by ultrasound [20,21]. Both assessments were repeated 3 times and averaged. The task was performed at baseline and one week after discharge.

The excursion was measured by elevating the patient’s trunk from the horizontal position by 10 to 15 degrees and during deep breathing. A curved probe (2–5 MHz) of the ultrasound machine (ACUSON NX3 Elite, Siemens Healthineers, Germany) was placed between the mid-clavicular line and anterior axillary line below the right costal margin to determine the line of the diaphragm in M-mode and the displacement of the right diaphragm was measured during the respiratory phase [20,21]. Diaphragm thickness was measured at the end-inspiratory and end-expiratory phases by elevating the patient’s trunk 10 to 15 degrees from the horizontal position during breathing (from FRC to TLC) using a linear probe (4–12 MHz) placed at ZOA . The diaphragm thickening fraction (TF) was calculated as the difference between thickness at end-inspiration and end-expiration. TF= (end-inspiratory thickness – end-expiratory thickness)/end-expiratory thickness × 100, which is an index of muscle shortening during contraction [8,20–24].

#### 2.4.5. Pulmonary function test (PFT)

Spirometry (microQuark, COSMED, Italy) is the most basic and useful form of pulmonary function test (PFT) that involves the measurement of exhaled or inhaled air during forced manoeuvres. Spirometry is a measurable, reproducible, non-invasive, and relatively simple method for measuring lung function. It can potentially identify obstructive or restrictive defects [25–27]. The most useful parameters for spirometry are FEV_1_, FVC, and the calculated ratio between them (FEV_1_/FVC). The measurements were based on the guidelines of the ATS and ERS. The standard method was to let participants take a sitting posture wearing a nose clip, bite the mouthpiece and hold the spirometer with one hand while the other hand fixes the lips with fingers to prevent air leakage. There are four phases of the FVC and FEV_1_ manoeuvre for spirometers that measure inspiration and expiration. First, the participants had to perform 3 tidal volume breaths as preparation. Second, they must inhale rapidly and entirely for a maximal inspiration breathing. Third, they must exhale a “blast” of expiration in the first second and continue to complete expiration for 6 s. Finally, they must inhale back to maximum lung volume by the maximal flow. The participants repeated the measurements 3 times, taking the maximum value as a record. The test was performed at baseline, pre-operation, post-operation, and one week after discharge [28].

#### 2.4.6. Quality of life

The World Health Organization Quality of Life Briefing (WHOQOL-BREF) was used for quality of life (QoL) assessment [29,30]. The test includes the categories of physical health, psychological state, social relationships, and environment and is translated into different languages in many countries [29,31].

### 2.5. Statistical analysis

The sample size was calculated based on the percentage difference in MIP between the IMT group and the CTL group. The sample size calculation was based on a standardized large effect size (*d* = 0. 80) with a power of 0.80 and alpha of 0.05. A total of 30 subjects were enrolled in this study. Data are presented as the mean ± standard deviation. Because of the small sample size in this study, all data were analysed by using nonparametric statistics. The Wilcoxon signed-rank test was used to detect differences at each time point within the group. The Mann–Whitney U test was used to detect differences between the CTL and IMT groups, with the significance level set as *p* < .05. All data analysis was performed by SPSS software version 22.0 (Ins., Chicago, IL, USA).

## 3. Results

### 3.1. Inspiratory muscle training (IMT) reduces the postoperative pulmonary complication (PCC) rate in patients undergoing upper abdominal surgery

**Figure 2.**
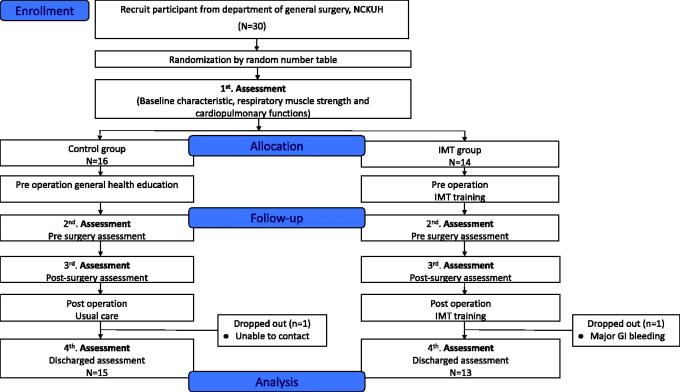
The flow chart of participant recruitment.

A total of 30 patients participated in this study, but 2 subjects were excluded from analysis. One subject withdrew because of changes in the treatment plan, and 1 subject was lost to follow-up. All participants completed 4 weeks of the training program and data collection (Figure 2). Participant demographics are shown in Table 1. All participants had undergone upper abdominal surgery (13 subjects received liver transplantation, and 15 subjects received surgery as the donor). Eighteen out of 28 participants were male; 10 of 28 participants were female. There were no statistically significant differences in age, sex, height, weight, diagnosis, or ASA scores between the CTL and IMT groups. According to the definition of PPCs in European Perioperative Clinical Outcome 2015, we found that the PPC rate of the CTL group was higher than that of the IMT group (CTL vs. IMT: 19 vs. 4, *p* < .001) during hospitalization; however, there was no statistically significant difference in the length of hospitalization (CTL vs. IMT: 18.5 vs. 21.5, *p* = .394).

**Table 1.** Demographic and clinical characteristics of the control group and IMT group.

	CTL group (*n* = 15)	IMT group (*n* = 13)	*p* value
Age	44.8 ± 14.2	46.4 ± 12.9	.609
Gender			
Male	11	7	–
Female	4	6	–
Height (cm)	165.3 ± 9.1	164.2 ± 7.7	.512
Weight (kg)	61.8 ± 9.4	68.4 ± 12.1	.12
Diagnosis			
Liver donor	8	6	–
Liver cirrhosis	7	7	–
ASA score	2.3 ± 2.3	2.2 ± 1.0	.939
MV days	1.2 ± 0.9	1.4 ± 1.6	.605
ICU days	8.6 ± 5.5	8.6 ± 5.3	.883
Admission days	18.5 ± 9.4	21.5 ± 12.0	.394
Length of surgery (mins)	568.2 ± 163.2	599.4 ± 156.4	.382
PPCs			
*R’t pleural effusion*	6	3	
*L’t pleural effusion*	4	0	
R’t Atelectasis	4	1	
L’t Atelectasis	3	0	
Pneumonia	1	0	
Pulmonary edoema	1	0	
*Any of above PPCs*	19	4	<.001*

Data were presented as mean ± SD. IMT: Inspiratory muscle training; ASA: American Society of Anaesthesiologists; PPCs: Postoperative pulmonary complications.

*p < .05 between group comparison.

### 3.2. IMT increases maximal inspiratory pressure (MIP) in patients with upper abdominal surgery

The MIP increased significantly compared to the baseline (Timepoint 1 vs. Timepoint 2: −124.7 vs. 170.2, *p* = .004) in the IMT group. In addition, there was a statistically significant difference between the CTL and IMT groups (CTL vs. IMT: −104.6 vs. −170.2, *p* = .013) at Timepoint 2. In terms of MEP performance, the CTL and IMT groups both showed the same degree of variability. The postoperative values decreased in comparison with the baseline and preoperative values, and the differences were statistically significant. The analysis showed that MIP increased significantly with the IMT program before surgery for at least two weeks and during the recovery period in the IMT group (Table 2).

**Table 2.** Respiratory muscle strength in the CTL and IMT group.

Respiratory muscle strength	Control group (*n* = 15)				IMT group (*n* = 13)			
	Timepoint 1	Timepoint 2	Timepoint 3	Timepoint 4	Timepoint 1	Timepoint 2	Timepoint 3	Timepoint 4
MIP (cmH2O)	–99.8 ± 40.4	–104.3 ± 49.8*	–86.2 ± 31.4	–109.6 ± 43	–124.7 ± 72	–170.2 ± 68.8^a,^*	–126.8 ± 63^d^	–151.5 ± 75^c,f^
MEP (cmH2O)	83.9 ± 42.5	69.8 ± 22.2	54.2 ± 40.3^b,d^	69.5 ± 24.2^e^	99.8 ± 52	107.1 ± 56.6	65.7 ± 35.1^b,d^	98.5 ± 44^e^

Data were presented as mean ± SD. Nonparametric analysis – Wilcoxon sign rank test was used for within-group analysis and the Mann-Whitney U test was used for analysis between groups.

IMT: Inspiratory muscle training; MIP: Maximal inspiratory pressure; MEP: Maximal expiratory pressure.

^a^*p* < .05 intragroup comparison between Timepoint 1 and Timepoint 2. ^b^*p* < .05 intragroup comparison between Timepoint 1 and Timepoint 3; ^c^*p* < .05 intragroup comparison between Timepoint 1 and Timepoint 4; ^d^*p* < .05 intragroup comparison between Timepoint 2 and Timepoint 3; ^e^*p* < .05 intragroup comparison between Timepoint 3 and Timepoint 4; *p < .05 between group comparison.

### 3.3. IMT did not affect cardiopulmonary function in patients who underwent upper abdominal surgery

The baseline CPET was comparable between the CTL and IMT groups. However, the performance of the IMT group in VO_2_ did not increase significantly at the anaerobic threshold (AT) or the maximal value after the IMT. However, the CTL group showed a significant difference in maxVO_2_ (*p* = .008), maxVO_2_HR (*p* = .006), and maxVEVO_2_ (*p* = .013) between Timepoint 1 and Timepoint 4. We found that the results suggested that a decrease in exercise capacity was observed in the CTL group without IMT intervention (Table 3).

**Table 3.** CPET in the CTL and IMT group.

	Control group (*n* = 15)			IMT group (*n* = 13)		
CPET	Timepoint 1	Timepoint 4	*p* value	Timepoint 1	Timepoint 4	*p* value
Anaerobic threshold (AT)						
Time (s)	430.1	345.3	.011*	455.1	388.8	.064
Exercise time (s)	355.9	275.1	.002*	381.4	323.2	.019
Work (watt)	88.7	54.9	.002*	94.6	64.8	.003*
V_T_	1.2	0.8	.004*	1.2	0.9	.002*
VE	23.7	18.2	.004*	27.6	21.2	.003*
VO_2_	14.1	9.8	.004*	13.7	10.8	.043*
VO_2_ml	909.1	597.1	.003*	963.1	683.8	.007*
VCO_2_VO2HR	888.2	578	.004*	944.2	673	.005*
VCO_2_HR	7.7	5.1	.003*	8.7	5.9	.003*
VE/VO_2_	28.4	32.5	.038*	29.3	31.4	.142
PTEO_2_	103.5	107.9	.018*	107.2	109.2	.365
Maximal value						
Work (watt)	141.1	90.2	.009*	125.1	101.6	.011*
VT	1.5	1.2	.004*	1.5	1.3	.059
RR	26.3**	31.9	.03*	33.5**	33.4	.889
VO_2_	19.6	16.1	.008*	19.6	17.9	.133
VO_2_ml	1271.4	977.9	.004*	1374	1116.6	.023*
VO_2_HR	9.4	7	.006*	10.2	7.8	.006*
VE/VO_2_	31.7	38.6	.013*	35.7	38.8	.263

Data were presented as mean ± SD. Nonparametric analysis – Wilcoxon sign rank test was used for within-group analysis and the Mann-Whitney U test was used for analysis between groups.

*p < .05 intragroup comparison; **p < .05 between group comparison.

AT: aerobic threshold; V_T_: tidal volume; VE: minute ventilation; VO_2_: oxygen consumption; VCO_2_: carbon dioxide output; VE/VO_2_: ventilator equivalent for oxygen; PTEO_2_: end-tidal pressure oxygen; RR: respiratory rate.

### 3.4. Effects of IMT on pulmonary function

Table 4 reveals that IMT prevented the decrease in FVC (%pred) at Timepoint 4 in patients who underwent upper abdominal surgery. IMT mitigated the decrease in FEV1 (%pred) in patients who underwent upper abdominal surgery at Timepoint 3. However, most results from the pulmonary function test indicated that IMT did not affect pulmonary function in patients with upper abdominal surgery.

**Table 4.** Pulmonary function test in the CTL and IMT group.

Pulmonary function test	Control group (*n* = 15)				IMT group (*n* = 13)			
	Time point 1	Time point 2	Time point 3	Time point 4	Time point 1	Time point 2	Time point 3	Time point 4
FVC (L)	3.4 ± 1.2	3.2 ± 1.3	1.4 ± 0.4^b,d^	2.7 ± 0.8^c,e,f^	3.1 ± 0.7	3.0 ± 0.8^a^	1.6 ± 0.5^b,d^	2.7 ± 0.6^f^
FEV1 (L)	2.8 ± 0.9	2.6 ± 1.1	1.1 ± 0.3^b,d^	2.3 ± 0.6^c,e,f^	2.5 ± 0.5	2.4 ± 0.6	1.3 ± 0.4^b,d^	2.2 ± 0.4^f^
FEV1/FVC (%)	84.3 ± 4.5	80.7 ± 9.9	79.4 ± 9.1	85.5 ± 5.6^f^	82.4 ± 3.2	82.4 ± 4.8	81.3 ± 4.8	82.3 ± 4.6
FVC (%pred)	87.3 ± 18.5	84.7 ± 23.7	37.3 ± 7.9^b,d^	70.2 ± 10.5^c,e,f,^*	89.7 ± 15.5	87.4 ± 20.5^a^	45.8 ± 14^b,d^	80.2 ± 10^c,e,^*
FEV1 (%pred)	87.7 ± 16.2	83.6 ± 24	35.9 ± 8.8^b,d,^*	71.5 ± 9.5^c,e,f^	88.3 ± 14	86.1 ± 20	44.8 ± 13.4^b,d,^*	78.8 ± 9.1^f^
FEV1/FVC (%pred)	100.9 ± 5.4	96.9 ± 11.6	96.1 ± 12	102.1 ± 5.8	98.6 ± 3.6	98.8 ± 4.8	97.5 ± 7.1	98.7 ± 5.2

Data were presented as mean ± SD. Nonparametric analysis – Wilcoxon sign rank test was used for within-group analysis and the Mann-Whitney U test was used for analysis between groups.

^a^*p* < .05 intragroup comparison between Timepoint 1 and Timepoint 2; ^b^*p* < .05 intragroup comparison between Timepoint 1 and Timepoint 3; ^c^*p* < .05 intragroup comparison between Timepoint 1 and Timepoint 4; ^d^*p* < .05 intragroup comparison between Timepoint 2 and Timepoint 3; ^e^*p* < .05 intragroup comparison between Timepoint 2 and Timepoint 4; ^f^*p* < .05 intragroup comparison between Timepoint 3 and Timepoint 4; *p < 0.05 between group comparison.

IMT: Inspiratory muscle training; FVC: Forced vital capacity; FEV1: Forced expiratory volume in 1 s.

### 3.5. Effects of IMT on diaphragm function and quality of life (QoL)

**Figure 3.**
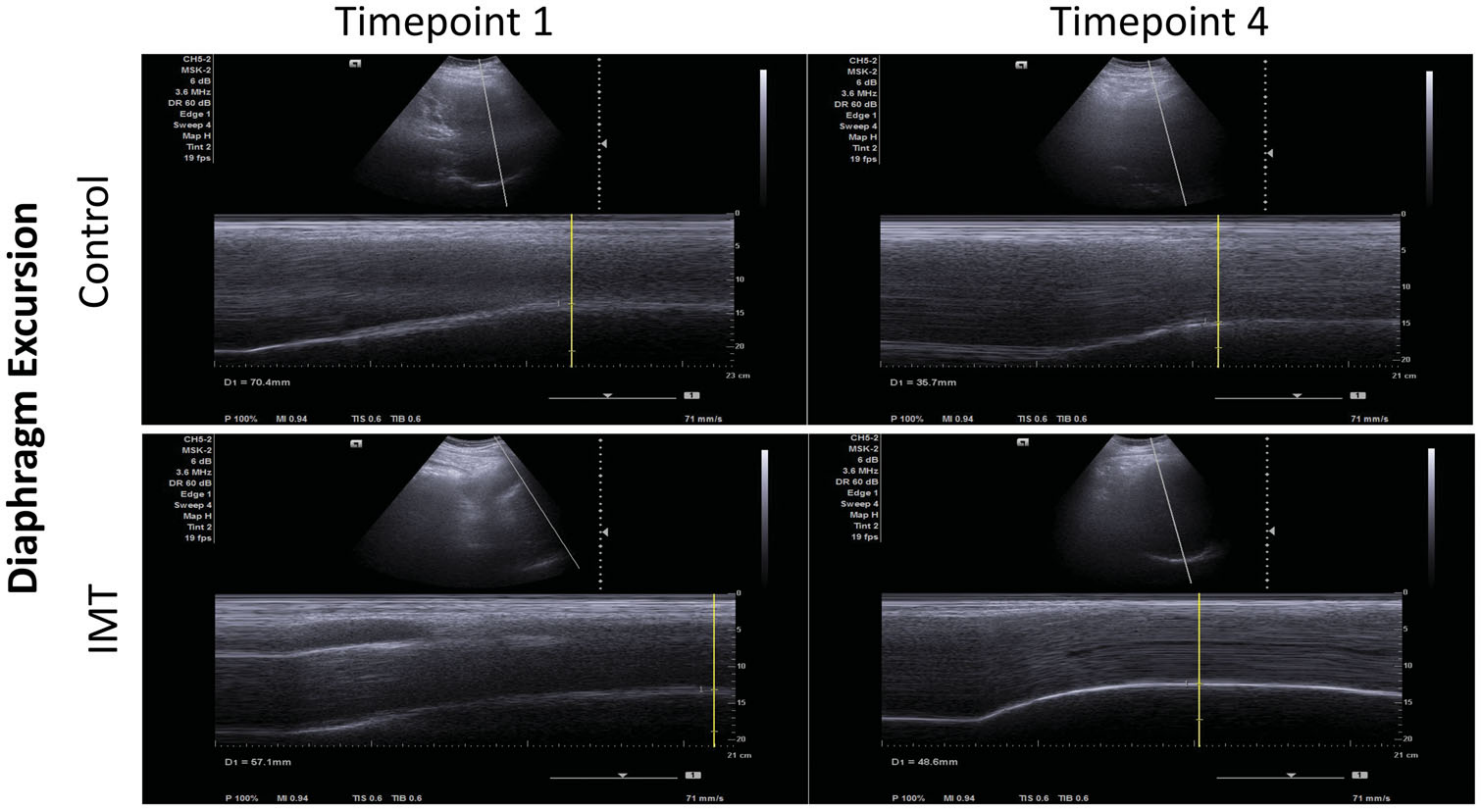
The M-mode images of diaphragmatic excursion in the CTL group and the IMT group at Timepoint 1 and Timepoint 4.

**Figure 4.**
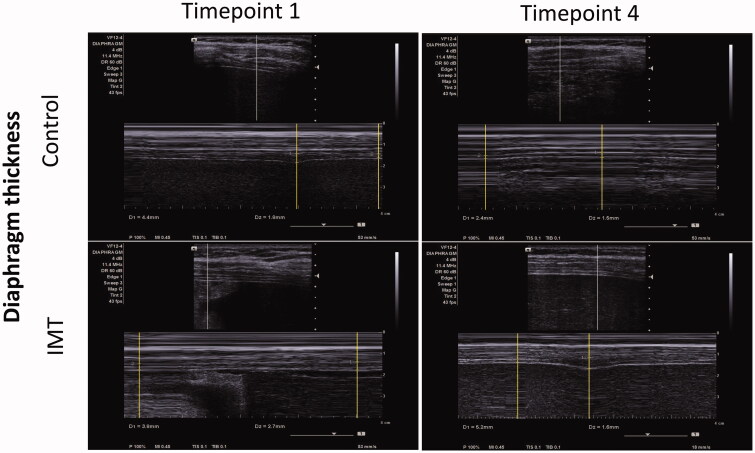
The M-mode images of the diaphragm thickening fraction in the CTL group and the IMT group at Timepoint 1 and Timepoint 4.

Next, we investigated whether IMT affects diaphragm function in patients who underwent upper abdominal surgery. Due to the surgery wound, the diaphragm ultrasonography could be performed only at Timepoint 1 and Timepoint 4. Table 5 shows that the baseline values of the diaphragmatic excursion were not significantly different between the CTL group and the IMT group. The CTL group showed a significant decline in the baseline values one week after discharge (Timepoint 1 vs. Timepoint 4: 52.0 vs. 25.3, *p* = .002). The IMT group preserved the capability of diaphragmatic excursion after IMT intervention (*p* = .06). At Timepoint 4, the diaphragmatic excursion was significantly higher in the IMT group than in the CTL group (CTL vs. IMT: 25.3 vs. 37.5, *p* = .012). The M-mode images of the diaphragmatic excursion are shown in Figure 3. Regarding the diaphragm thickening fraction, there was no significant difference between the CTL group and the IMT group, either within or between the groups. The M-mode images of the diaphragm thickening fraction are shown in Figure 4.

**Table 5.** Diaphragm ultrasonography measurements and WHOQOL-BREF scores in the CTL and IMT group.

	Control group (*n* = 15)			IMT group (*n* = 13)			T1 between group *p* value	T4 between group *p* value
	Timepoint 1	Timepoint 4	*p* value	Timepoint 1	Timepoint 4	*p* value		
Diaphragm ultrasonography								
Diaphragmatic excursion (mm)	52.0 ± 26.5	25.3 ± 8.8	.002*	52.9 ± 18.2	39.3 ± 13.5	.06	.593	.012**
Diaphragm thickening fraction (%)	77.6 ± 67.1	51.7 ± 39.1	.198	67.9 ± 30.5	59.6 ± 31.3	.463	.734	.174
WHOQOL-BREF scores								
Physical health domain	25 ± 3.6	22.8 ± 4	.045*	23.6 ± 6.4	24.1 ± 3.5	.844	.608	.464
Psychological domain	20.6 ± 3.3	20.6 ± 4	.665	20.5 ± 4	21.1 ± 2.6	.327	.981	.922
Social relationships domain (Taiwan)	13.8 ± 2	13.9 ± 1.8	.666	14.3 ± 1.8	14.2 ± 1.6	.999	.521	.633
Environment domain (Taiwan)	30.8 ± 3.4	31.6 ± 3.1	.823	32.7 ± 4	33.7 ± 3.4	.999	.622	.617

Data were presented as mean ± SD. Nonparametric analysis – Wilcoxon sign rank test was used for within-group analysis and the Mann-Whitney U test was used for analysis between groups.

*p < .05 intragroup comparison; **p < .05 between group comparison.

Time point 1 & T1: baseline, Time point 4 & T4: one week after discharge.

There was no significant difference in the performance before and after the surgery in the psychological domain, social relationships domain, and environment domain of WHOQOL-BREF between the groups after undergoing respiratory muscle training encompassing from preoperative to postoperative periods. However, we found a significant decrease in the physical health domain of WHOQOL-BREF after the termination of the trial in the CTL group, while there was a modest elevation in the IMT group (CTL: 25 vs. 22.78, *p* = .045) (Table 5).

## 4. Discussion

The term “PPCs” involves most of the complications that affect the respiratory system after anaesthesia in surgery and adverse effects during postoperative recovery [2]. The frequency of clinically relevant postoperative pulmonary complications following upper abdominal surgery ranges from 1 to 30% of the patients [1]. This is the first RCT study that indicated that fully engaged IMT improves respiratory muscle strength and diaphragmatic excursion. Fully engaged IMT also has a beneficial effect on the incidence of PPCs compared to the CLT group.

We found a marked improvement in MIP with a significant difference and a lower incidence of PPCs in the IMT group who received 3 weeks of preoperative training compared to the CTL group. Such results are consistent with previous experimental results in IMT interventions in surgical patients [15,32]. Further analysis revealed that our participants underwent liver transplantation, and pleural effusion was one of the common pulmonary complications after liver transplantation, especially on the right side [33]. Pleural effusion after hepatic surgery is believed to be mostly due to anatomical defects, where peritoneal fluid leaks through the diaphragm. Damage to the right diaphragm may cause transection of the hepatic lymphatic vessel, and blood transfusion during surgery and hypoalbuminemia may contribute to this problem. This would explain why right pleural effusion occurs in post-liver transplantation patients. These are not the common lung collapse of PPCs [33,34]. Even though hepatic pleural effusion is one of the most common PPCs after liver transplantation, we observed that the rate of complications in the extended intervention IMT group was significantly lower than that in the CTL group. This is consistent with the results of a comprehensive analysis of cardiothoracic and abdominal surgery; IMT can improve inspiratory muscle strength and reduce the rate of PPCs.

The diaphragm is the most important respiratory muscle and plays a major role in maintaining ventilation to the respiratory system. There are several clinical methods of monitoring diaphragmatic function. Diaphragm ultrasonography has played a crucial role in evaluating many aspects of critical illness and has recently been proposed as a tool to quantify the diaphragm [8]. We found that the CTL group showed a significant decrease in the diaphragmatic excursion at the end of the experiment compared to the baseline. There was a significant difference between the groups. Therefore, it could be seen that the diaphragm’s mobility is better preserved in the IMT group despite the physical damage of the surgery, and it can be inferred that the IMT program can help the recovery of the diaphragm after the operation.

Pulmonary function provides a clinical basis for decision-making for patients with lung disease and provides detailed information [25]. In terms of pulmonary function performance, it was found that the FEV_1_ and FVC measured at various time points in both the CTL and IMT groups were not significantly different. The lung volumes in both groups did not change with training and showed similar decreases after surgery, suggesting that IMT will not affect these variables. Dronkers et al. also found that although threshold IMT improved MIP, it did not affect lung capacity [35]. Such results indicated that inspiratory muscle function recovered more rapidly in the IMT group after surgery. However, this improvement did not affect lung capacity after surgery [36].

The limitations of this study are the small sample size, the single surgical procedure, and the lack of long-term follow-up. Due to the epidemiological impact of COVID-19 during the enrolment period of this study, the participants’ willingness was reduced, and the implementation of long-term follow-up was restricted. The type of surgical procedures may affect the effectiveness of this training modality.

### Interpretation

This study suggested that the fully engaged IMT intervention program improves respiratory strength, diaphragmatic mobility, and QoL and reduces the incidence of PPCs in patients undergoing upper abdominal surgery.

## Guarantor statement

Dr. Kun-Ling Tsai assumes responsibility for being the guarantor, taking responsibility for the integrity of this study.

## Ethics approval

This study has been approved by the National Cheng Kung University Hospital Institutional Review Board, Tainan, Taiwan (B-BR-108-012), and this trial was registered in the Thai Clinical Trials Registry (TCTR20190526001) and ClinicalTrials.gov (NCT05239819).

## Author contributions

All authors contributed to the conception or design of the study. Yu-Ting Huang, Yih-Jyh Lin. and Kun-Ling Tsai conceived of, designed, and supervised the study. Yu-Ting Huang, Ching-Hsia Hung, and Hui-Ching Cheng performed the experiments and analysed the data. Hui-Ching Cheng, Hsin-Lun Yang, and Yi-Fang Tsai assisted with the experiments and data collection. Yi-Liang Kuo and Pei-Ming Chu reviewed and edited this submission. Yu-Ting Huang and Kun-Ling Tsai wrote the manuscript. Yu-Ting Huang and Kun-Ling Tsai contributed to the final version of the manuscript. All authors have read and agreed to the published version of the manuscript.

## Supplementary Material

Supplemental Material

## Data Availability

The data presented in this study are available in the article.
